# Transmission of infectious laryngotracheitis virus vaccine and field strains: the role of degree of contact and transmission by whole blood, plasma and poultry dust

**DOI:** 10.1186/s13567-021-00959-1

**Published:** 2021-06-22

**Authors:** Addisu A. Yegoraw, Awol M. Assen, Priscilla F. Gerber, Stephen W. Walkden-Brown

**Affiliations:** 1 Animal Science, School of Environmental and Rural Science, University of New England, Armidale, NSW Australia; 2 School of Veterinary Medicine, Wolaita Sodo University, Wolaita Sodo, Ethiopia; 3 School of Veterinary Medicine, Wollo University, Dessie, Ethiopia

**Keywords:** Infectious laryngotracheitis virus, Transmission, Contact, Airborne, Dust, Meat chickens, Vaccine

## Abstract

Understanding the mechanisms of transmission of infectious laryngotracheitis virus (ILTV) is critical to proper control as both vaccine and wild-type strains circulate within chicken flocks with potential adverse consequences. The relative efficiency of transmission by direct contact between chickens and airborne transmission has not been investigated. Furthermore, relatively high levels of ILTV DNA have been detected in poultry dust and blood but the infectivity of these is unknown. In this study, comparison of in-contact and airborne transmission of two vaccine and one field strain of ILTV revealed that all transmitted to 100% of in-contact birds by 6 days post-exposure (dpe). Airborne transmission without contact resulted in 100% transmission by 14 and 17 dpe for the wild-type and Serva vaccine virus but only 27% transmission by 21 dpe for the A20 vaccine virus. The infectivity of dust or extracts of dust and blood or plasma from infected chickens at various stages of infection was assessed by inoculation into susceptible chickens. There was no transmission by any of these materials. In conclusion, direct contact facilitated efficient ILTV transmission but the virus was unable to be transmitted by dust from infected chickens suggestive of a limited role in the epidemiology of ILTV.

## Introduction

Avian infectious laryngotracheitis (ILT) is an important and widespread respiratory and ocular disease of chickens [1]. The disease is caused by infectious laryngotracheitis virus (ILTV), a virus belonging to the subfamily *Alphaherpesvirinae* of the family *Herpesviridae* [2]. The disease chiefly affects the conjunctiva and tracheal mucosa resulting in inflammation, serous or mucous discharge, haemorrhagic in severe cases, coughing and dyspnoea associated with tracheal necrotic plugs, accompanied by decreased egg production and/or weight gain [1]. The disease has variable morbidity (up to 90%) and mortality (up to 70%) rates [1, 3, 4], depending on the virulence of the circulating strains [5].

In Australia, ILT remains a disease of significant concern to the broiler chicken industry due to a prolonged outbreak in high producing areas in Victoria and New South Wales (NSW) that has not been brought under adequate control, despite the availability and use of live vaccines [6, 7]. In Australia only live attenuated vaccines are available, these being Australian chick embryo origin (CEO) vaccines SA2 and A20 (SA2 strain further attenuated by 20 passages in cell culture and then 5 passages in embryonated eggs) [8] and European strain Serva. Depending on the vaccine strains and age of birds infected, live attenuated vaccine strains particularly CEO strains have the ability to produce clinical signs, tracheal lesions and mortality like the field strains [9–12]. Live attenuated vaccines may also show reversion to virulence following passage between birds after vaccination [13] and in Australia have been responsible for the emergence of new virulent strains due to natural recombination between them [14] with associated increased replication rate, infectivity and enhanced transmissibility to in-contact birds of some recombinants [15].

ILTV can be transmitted horizontally through the respiratory, conjunctival or to a lesser extent oral routes [16–19]. Recent studies have demonstrated that live ILT vaccines transmit readily from infected to susceptible in-contact chickens experimentally [20–22] and in the field [6]. Direct physical contact between chickens may be important in transmission of ILTV as infected birds have a high incidence of conjunctivitis following infection by eye drop [23], or airborne transmission [24]. Affected birds typically show excessive lachrymation and tear-staining of feathers accompanied by huddling together which would facilitate contact transmission. The virus also transmits readily between farms [7, 25] by indirect transmission via carriers of freshly excreted virus through contaminated equipment, clothing, trucks, and litter [26–28]. Wind borne transmission is also implicated in transmission between farms [25, 29–31]. We have recently reported poor airborne transmission of vaccine strains of ILTV (A20, SA2 and Serva) relative to virulent field strains [24] but the importance of direct physical contact between chickens in transmission within flocks has not been investigated to date.

ILTV DNA is readily detected in poultry dust from ILTV infected chickens [32–35] and dust is implicated in the epidemiology of ILT with outbreaks of the disease thought to be associated with spread of spent litter from contaminated poultry farms [25]. This is reinforced by experimental demonstration of airborne transmission of field and vaccine ILTV strains, potentially due to infective dust particles in the air [24]. In contrast, Bindari et al. [36] were unable to isolate ILTV from ILTV PCR positive dust samples in either chick embryos or cell culture. Thus the role of poultry dust in the transmission of ILTV remains unresolved.

ILTV DNA can be detected in many organs of infected chickens outside of the respiratory tract [34, 37–40] including blood of infected chickens where it is concentrated in the leucocyte and plasma fractions [40]. While some studies have reported the lack of a viraemic phase of ILTV infection [16, 17] in vitro studies have shown replication of ILTV in macrophages and buffy coat cells [41, 42]. If indeed infective virus is present in the bloodstream, virus transmission by haematophagous insects such as mosquitoes or sucking lice and mites is a possibility as is the case with diseases such as fowlpox [43]. Importantly, ILTV has been shown to survive in another insect pest the darkling beetle (*Alphitobius diaperinus*) for up to 42 days following an outbreak of ILTV, implicating it in the transmission of ILTV [44].

In light of the above, three experiments were designed to test the following propositions: (1) field and vaccine ILTV strains will transmit much more effectively to in-contact susceptible birds than those sharing an air space without direct contact; (2) Direct application of fresh dust or extracts of fresh dust from chickens with active infection with virulent ILTV will transmit the virus to susceptible chickens and (3) Inoculation of fresh whole blood and plasma from chickens with active ILTV infection will transmit the virus to susceptible chickens.

## Materials and methods

In order to test the propositions three experiments were carried out concurrently in a containment Level 2 isolator facility containing 21 isolators.

### Birds, housing and management

The experiments were approved by the University of New England (UNE) Animal Ethics Committee (AEC19-102). A total of 344 newly hatched unsexed commercial Ross broiler chicks were used in a containment Level 2 isolator facility containing 21 isolators. Each isolator has a floor space of 1.35 m^2^ and the chicks were placed on pine shavings bedding material within the isolator. Commercial broiler starter then grower feed and water were available ad libitum. Air inlet temperatures were set at 35 °C initially declining by 1 °C every other day until 21 °C was reached. Individual chicks were identified with numbered padlock style wing tags applied at placement in the isolators. Birds were monitored for well-being twice daily.

### Virus strains

Two live commercial ILTV vaccines and one field strain were used in these experiments. The vaccines were Class 1 strain A20 (Poulvac Laryngo A20, Zoetis, Australia) and Class 7 strain Serva (Nobillis ILT, MSD, Australia). The vaccines were administered at twice the minimum recommended dose by the manufacturers (one dose in each eye). A Class 9 strain NSW/18 B2 was isolated from tracheal tissues obtained from a 2017 ILTV outbreak in commercial meat chicken farms in NSW, Australia. Class 9 was propagated in LMH (Leghorn Male Hepatoma, ATCC) cells for three passages at UNE as previously described [23]. The median tissue culture infective dose (TCID_50_) titre was estimated by the method of Reed and Muench [45].

### Experimental design, treatment application and measurements

Details of the three experiments, including bird ages are summarized in Table 2 and described in detail below.

#### Experiment 1. Degree of contact transmission (CONTACT)

To test the transmission of Class 9 and vaccine (Serva and A20) ILTV strains between birds with different degrees of contact, pairs of isolators were modified such that they had a shared airspace, but direct contact between birds in each isolator was not possible. This was achieved by enclosing a pair of isolator frames in a single isolator wrap with a solid barrier extending along the length of the joined isolators and rising to 2/3 of their height, with a wire mesh barrier above this to enable air exchange. Half of the chicks (*n* = 8) in the first isolator of each pair were inoculated at 7 days of age with the relevant vaccine or challenge virus while the other 8 chicks remained in-contact with the inoculated chicks. The eye drop inoculated birds were segregated for 8 h before mixing with the in-contact birds. Sixteen chicks in the second isolator shared airspace with the first group but had no direct contact with infected birds. HEPA filtered inlet air was ducted into the first isolator containing the eye drop infected donor birds and ducted out from the second isolator containing the shared airspace birds. Shared airspace birds were exposed to inoculated birds commencing on the day of infection (7 days of age). To assess infection and transmission of the ILTV strains choanal cleft swabs were collected on 3, 6, 10, 14, 17 and 21 days post-exposure (dpe). Brachial vein blood was collected from the eye drop inoculated birds of all groups on 6, 10, 14 and 21 dpe. The blood collected on 6 and 10 dpe was used as inoculum for experiment 3 (BLOOD INF). Details of the treatments are provided in Tables 1 and 2.

**Table 1:** **Details of the dose and batch number of ILTV used for the different groups**

ILTV strain	Classification	Batch number	Dose administered by eye drop
NSW/18 B2 (Class 9)	Virulent wild-type	B2P3_20180614	10^4^TCID_50_/bird and 10^3^TCID_50_/bird
Serva	Vaccine	B.1707904	10^2.8^ EID/bird
A20	Vaccine	B3.44135	10^3.5^ PFU/bird

EID: embryo infective dose, PFU: plaque forming unit, TCID: tissue culture infective dose.

**Table 2:** **Details of the experiments: dose, mode of exposure and type of inoculum used in each experiment**

Experiment	Treatment	Isolator (n)	Chickens/isolator (n)	ILTV strain	Inoculum	Mode of exposure	Dose	Age at exposure (d)
Expt. 1 CONTACT	Eye drop (donors)	3	8	A20 Serva C9	Virus	Eye drop	^a^10^3.5^ PFU/bird (A20) 10^2.8^ EID/bird (Serva) 10^4^TCID_50_/bird (Class 9)	14
	In-contact	3	8	A20 Serva C9	Nil	Contact with donors		
	Shared air space	3	16	A20 Serva C9	Nil	Shared air with donors		
Expt. 2 DUST INF	3 dpe dust	2	15	C9	Dust or dust extract	Eye/URT^b^	60 µL extract, 5–10 mg dust	10
	7 dpe dust	2	15	C9				14
	14 dpe dust	2	15	C9				21
Expt. 3 BLOOD INF	A20	1	15	A20	Plasma + fresh whole blood^d^	Eye/intra coelemic^c^	60 µL plasma + 1 mL blood	20 & 24
	Serva	1	15	Serva				
	Class 9	1	15	C9				
Control	Normal saline	2	16–17	-	Normal saline	Eye drop	-	7

C9, class 9; URT, upper respiratory tract.

^a^Dose for eye drop infection of donor birds only.

^b^Extract administered by eye drop (*n* = 15), dust insufflated into nares (*n* = 5), laryngopharyngeal space (*n* = 5) or trachea (*n* = 5).

^c^Plasma and whole fresh blood collected at 6 and 10 dpe of infected birds.

^d^Plasma administered by eye drop and whole fresh blood by intra-coelemic (“abdominal”) injection to the same chicken.

#### Experiment 2. Dust infectivity (DUST INF)

The experimental details for the dust infectivity experiment are summarized in Table 2. To generate infective dust containing virulent ILTV for use in this experiment, 20 chickens in each of 4 isolators were infected with 10^3^ TCID_50_ Class 9 at 7 days of age. Dust samples were collected from the isolator exhausts of these isolators as previously described [46] at 3, 7 and 14 dpe and used to challenge birds in 2 isolators for each dpe by eye-drop application of an aqueous extract of fresh dust or by direct insufflation of fresh dust. Birds were then challenged at 10, 14 and 21 days of age with dust collected at 3, 7 and 14 dpe, respectively. For each dpe 15 birds in one isolator were administered a dust extract by eye drop and in the other isolator birds were administered by direct insufflation of dust into the nares (*n* = 5), trachea (*n* = 5) or pharynx (*n* = 5). For preparation of the dust extract, approximately 150 mg of dust was mixed with 2.2 mL of Waymouth’s sterile medium with 10% antibiotics (penicillin/streptomycin). After vortexing and centrifugation 1.7 mL of supernatant was recovered. Each bird was administered with 60 µL of this mixture, representing the extract of approximately 5.3 mg of dust. Quantitative PCR analysis of the inocula revealed that ILTV genome copies (GC) were 10^2.81^, 10^5.92^ and 10^5.62^ GC/µL of dust extract and 10^5.22^, 10^7.77^ and 10^6.54^ GC/mg of dust for the 3, 7 and 14 dpe inocula respectively. To assess in vivo infectivity of the inocula, choanal cleft swabs were collected on 7 and 14 dpe.

#### Experiment 3. Blood infectivity (BLOOD INF)

For this experiment 15 birds in each of 3 isolators were inoculated with fresh blood and plasma collected from birds infected with Class 9, Serva or A20 ILTV strains (eye drop inoculated birds from Experiment 1) at 6 and 10 dpe. Blood and plasma inocula were prepared as described in “Data recording and sampling methodology” section. Half of the chickens in each of the isolators received 60 µL of fresh plasma by eye-drop plus 1 mL of whole fresh blood into the intra-coelomic cavity at the age of 20 days (blood and plasma from 6 dpe) and the other half of the birds received the same treatment at the age of 24 days (blood and plasma from 10 dpe). Quantitative PCR analysis of the inocula revealed GC values for the 6 dpe plasma inocula of 10^4.44^ GC/mL (A20), 10^5.25^ GC/mL (Serva) and 0 GC/mL (Class 9), for blood inocula from 6 dpe, 0 GC/mL (A20 and Serva) and 10^3.91^ GC/mL (Class 9); for plasma inocula from 10 dpe, 10^3.35^ GC/mL (A20), 10^5.35^ GC/mL (Serva) and 10^4.2^ GC/mL (Class 9) and the blood inocula from 10 dpe was 0 GC/mL for all ILTV strains. The recipient birds were 20 and 24 days of age respectively when inoculated. To assess in vivo infectivity of the inocula choanal cleft swabs were collected on 7 and 14 dpe.

### Data recording and sampling methodology

Birds were monitored for well-being twice daily. After inoculation with virus or potentially infective materials, signs of disease such as depression, respiratory signs including sneezing, coughing, and gasping were recorded daily. Severely ill birds were euthanized for welfare reasons. All dead and euthanized chickens were subjected to post-mortem examination. Individual chickens were scored daily for clinical signs from 2 to 28 dpe using a scoring system modified from Kirkpatrick et al. [10]. Ocular signs were scored on a scale of 0 (none) 1 (unilateral conjunctivitis), 2 (bilateral conjunctivitis), 3 (partial closure of eye) and 4 (complete closure of the eye). Respiratory signs were scored from 0 (none) to 4 (severe gasping with neck extension). The overall demeanour of the chickens was scored from 0 (normal) to 2 (severely depressed). An overall clinical sign score was then calculated for each bird by summing together the scores for each clinical sign. Choanal cleft swabs were collected using sterile flocked swabs (FLOQSwabs, COPAN, Brescia, Italy). Blood was collected from the brachial (wing) vein using 3 mL sterile syringes and transferred to collection vials containing 3.2% sodium citrate anticoagulant (Expt. 1). Blood collected from Expt. 1 CONTACT (eye drop inoculated birds), on 6 and 10 dpe was pooled and the pooled blood divided into one tube for plasma separation and another tube for whole blood to use as inoculum for Expt. 3 BLOOD INF. Dust was collected from filter bags placed in the isolator exhaust ducts from 2–3 days prior to collection. Swab, dust and plasma samples were stored at -20 °C until analysis.

### Nucleic acid extraction and ILTV GC detection

Choanal cleft swabs were placed in 1.5 mL microtube that contains 0.8 mL of sterile phosphate buffered solution and vortexed for 10 s prior to nucleic acid extraction. DNA was extracted from approximately 5 mg of dust and/or 200 µL of choanal swab wash, and 200 µL of plasma using the Bioline ISOLATE II Genomic DNA kit [33]. DNA extracts were tested for the ILTV glycoprotein C gene using a Taqman® based qPCR assay [47] with absolute quantification using a standard curve based on a plasmid preparation of the target sequence. The DNA elution volume was 100 μL and 3 μL of this was used as the template in the qPCR reaction. Results were reported in log_10_ GC per milligram dust, per reaction for choanal cleft swab, and per millilitre of plasma.

### Determination that a chicken was infected with ILTV

Contamination of the choanal cleft of birds with inactivated ILTV is possible during dust bathing and other activity and this has the potential to produce false positive results in determining the presence of active infection. Based on considerable accumulated experience with this method and examination of the results of a number of experiments the following criteria were used to determine the presence of active infection (i) a positive ILTV choanal cleft swab for 2 consecutive samplings with ILTV DNA of 10^3^ GC/reaction or higher on at least one of those samples, or the bird died or was euthanised with signs of ILT, (ii) had a positive swab on the last sampling day accompanied by signs or lesions of ILT, or (iii) had a positive swab on the last sampling day with ILTV DNA of 10^5^ GC/reaction or higher irrespective of ILT signs. When a bird was identified as positive, the time of first positive is the date of first test in the series providing evidence of infection.

### Statistical analyses

Statistical analyses were performed with JMP v.14 software (SAS Institute, Cary USA). Discrete data (positive or negative for ILTV DNA) were subject to contingency table analysis, with significance between means determined by the Chi-square test of independence and where numbers in cells were below 5 by Fisher’s exact test. Patterns of infection over time were subjected to survival analysis using the nonparametric Kaplan–Meier method. ILTV GC were transformed into log_10_ before analyses (log_10_ GC + 1). This transformation means that negative samples are given a zero value in analyses of this variable. Repeated measures (ILTV GC and clinical scores) were assessed using a mixed restricted maximum likelihood model fitting individual bird as a random factor and treatments, dpe and their interactions as fixed effects. For measurements that were not repeated (e.g. total clinical score) linear models fitting the given treatment and their interactions was fitted to test significance of these effects. The significance of differences between means within a significant main effect were determined using Tukey’s HSD test. A significance level of *P* ≤ 0.05 was used throughout this study. For continuous variables, least squares means (LSM) and standard error means (SE) are presented.

## Results

The development of clinical signs (conjunctivitis, dyspnoea) and the detection of ILTV genome in choanal cleft swabs from eye drop inoculated birds (Class 9, A20 and Serva) indicates that viral infections were established successfully. None of the birds from the negative control groups were positive for ILTV infection indicating proper functioning of the isolators.

### Experiment 1. Degree of contact transmission

#### Clinical signs and mortality

Two birds died or were euthanized with clinical signs of ILT during the experiment, one from in-contact with Class 9 eye drop inoculated birds at 17 dpe and the other after 16 days of shared airspace with the Class 9 infected birds. The proportions of birds showing clinical signs and the severity of the clinical signs were significantly influenced by ILTV strain, mode of infection and dpe, and in the case of clinical scores, there was significant interaction between these treatment effects (Table 3). The most common clincal sign observed by far was conjunctivitis, irrespective of the mode of infection (Table 4). In severe cases this led to adherence of eyelids with inflammatory exudate and induced blindness of affected eyes. Respiratory signs and unwillingness to move were less frequent. However, respiratory signs, mainly dyspneoa with gasping were recorded in 2/8 and 2/15 of chickens in-contact with or having a shared airspace with chickens inoculated with Class 9 ILTV by eye drop respectively (Table 4).

**Table 3:** **Experiment 1 CONTACT. Summary of analyses of clinical scores and ILTV GC in choanal cleft swabs (LSM ±S.E) showing treatment effects and their significance**

Factors and levels	Clinical signs		qPCR result of choanal cleft swabs	
	N.positive/total (%)	Clinical score	N.positive/total (%)	Log_10_ GC/reaction
Overall	45/92 (49)	0.23 ± 0.04	81/92 (88)	4.23 ± 0.09
Factor and level				
Mode of exposure	***P* = 0.0013**	***P* < 0.0001**	***P* < 0.0019**	***P* < 0.0001**
Eye drop	14/23 (61)^a^	0.35 ± 0.05^a^	23/23 (100)^a^	5.02 ± 0.16^a^
In-contact	17/23 (74)^a^	0.39 ± 0.05^a^	23/23 (100)^a^	4.78 ± 0.16^a^
Shared airspace	14/46 (30)^b^	0.09 ± 0.04^b^	35/46 (76)^b^	2.89 ± 0.12^b^
ILTV Strains	***P* < 0.0001**	***P* < 0.0001**	***P* < 0.0001**	*P* = 0.09
A20	2/29 (7)^c^	0.02 ± 0.05^c^	18/29 (62)^b^	3.95 ± 0.16^a^
Serva	18/32 (56)^b^	0.27 ± 0.05^b^	32/32 (100)^a^	4.39 ± 0.15^a^
Class 9	25/31 (81)^a^	0.55 ± 0.05^a^	31/31 (100)^a^	4.35 ± 0.15^a^
dpe	*P* = 0.12	***P* < 0.0001**		***P* < 0.0001**
3	–	–		2.86 ± 0.16^b^
5	6/95 (6)	0.1 ± 0.05^c^		–
6	13/95 (14)	0.21 ± 0.05^bc^		4.65 ± 0.16^a^
7	13/95 (14)	0.21 ± 0.05^bc^		–
8	20/95 (21)	0.33 ± 0.05^ab^		–
9	21/95 (22)	0.44 ± 0.05^a^		–
10	16/95 (17)	0.28 ± 0.05^abc^		4.69 ± 0.16^a^
11	21/95 (22)	0.39 ± 0.05^ab^		–
13	15/95 (16)	0.29 ± 0.05^ab^		–
14	17/95 (18)	0.26 ± 0.05^abc^		4.35 ± 0.16^a^
15	21/95 (22)	0.32 ± 0.05^ab^		–
16	18/95 (19)	0.28 ± 0.05^abc^		–
17	16/95 (17)	0.26 ± 0.05^abc^		4.31 ± 0.16^a^
18	21/95 (22)	0.26 ± 0.05^abc^		–
21	23/95 (24)	0.29 ± 0.05^ab^		4.51 ± 0.16^a^
Interaction (*P*-value)				
ILTV strain*Dpe		***P* < 0.0001**		***P* < 0.0001**
ILTV strains*ME		***P* < 0.0155**		***P* < 0.0001**
Mode of exposure*Dpe		***P* < 0.0001**		***P* < 0.0001**
ILTV strains*ME*Dpe		***P* < 0.0001**		***P* < 0.0001**

Summary of analyses of clinical scores and ILTV GC in choanal cleft swabs (LSM ± S.E) showing treatment effects and their significance.

^*^dpe: days post-exposure, GC: genome copy, ME: mode of exposure.

^abc^Different letters within columns for each factor indicate significant differences between levels (*P* < 0.05). Bold text indicates statistically significant values (*P* < 0.05).

**Table 4:** **Experiment 1 CONTACT**

ILTV strains	Clinical sign	Proportion of chickens (%) showing ILT clinical signs by mode of exposure			
		Eye drop (%)	In- contact (%)	Shared airspace (%)	All modes of exposure
A20	Conjunctivitis	0/7 (0.00)	2/7 (28.6)	0/15 (0.00)	2/29 (7)
	Respiratory signs	0/7 (0.00)	0/7 (0.00)	0/15 (0.00)	0/29 (0.00)
	Demeanour	0/7 (0.00)	0/7 (0.00)	0/15 (0.00)	0/29 (0.00)
	Any clinical signs	0/7 (0.00)	2/7 (29)	0/15 (0.00)	2/29 (7)
Serva	Conjunctivitis	6/8 (75)^abA^	7/8 (87.5)^a^	5/16 (31.3)^b^	18/32 (56)^A^
	Respiratory signs	0/8 (0.00)^B^	0/8 (0.00)	0/16 (0.00)	0/32 (0.00)^B^
	Demeanour	0/8 (0.00)^aB^	1/8 (12.5)	0/16 (0.00)	1/32 (3)^B^
	Any clinical signs	6/8 (75)^ab^	7/8 (88)^a^	5/16 (31)^b^	18/32 (56)
Class 9	Conjunctivitis	8/8 (100)^aA^	8/8 (100)^aA^	7/15 (46.7)^bA^	23/31 (74)^A^
	Respiratory signs	0/8 (0.00)^B^	2/8 (25)^B^	2/15 (13.3)^B^	4/31 (13)^B^
	Demeanour	0/8 (0.00)^B^	1/8 (12.5)^B^	0/15 (0.00)^B^	1/31 (3)^B^
	Any clinical signs	8/8 (100)	8/8 (100)	9/15 (60)	25/31 (81)

Type and frequency of clinical signs observed over the full period of 21 dpe showing interaction between the effects of ILTV strain and mode of exposure.

^ab^Different lowercase superscripts indicate significant differences within rows.

^AB^Uppercase superscripts indicate a significant differences within columns for each ILTV strain.

**Figure 1:**
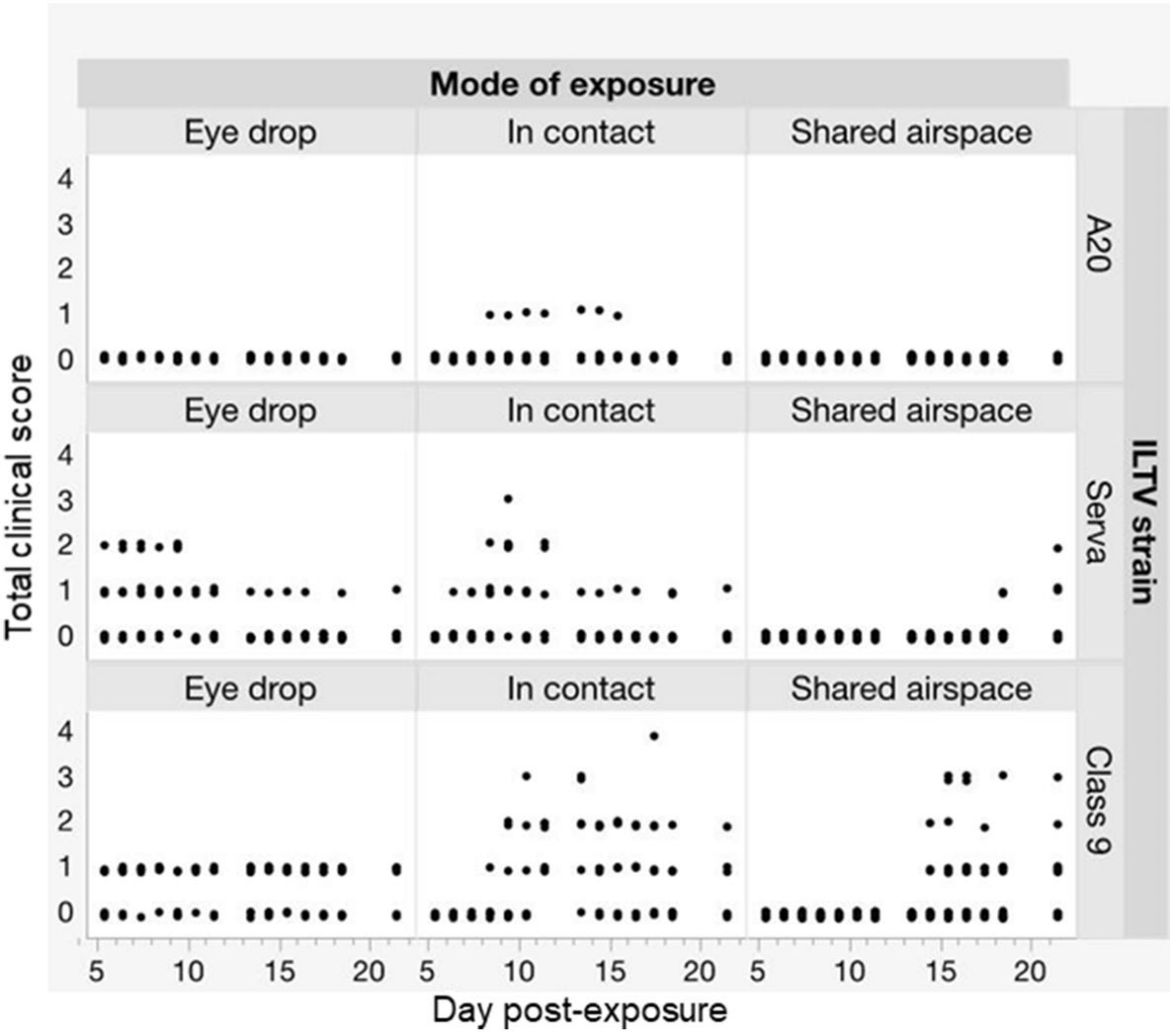
**Experiment 1 CONTACT.** Interaction between the effects of mode of infection, ILTV strain, and days post-exposure on the severity of clinical scores. Each point represents a chicken on a given dpe.

Visualisation of the interaction between treatment effects and time post-exposure on clinical signs shows that Class 9 virus induced the most severe and frequent clincal signs followed by Serva with A20 inducing a very low level of clinical signs only in in-contact birds (Figure 1). Clinical scores were highest for in-contact birds and commenced at 6–8 dpe, compared to 5 dpe in eye-drop infected birds and 14 dpe in birds with shared airspace.

#### Detection and quantification of ILTV genome copies in choanal cleft swabs

**Figure 2:**
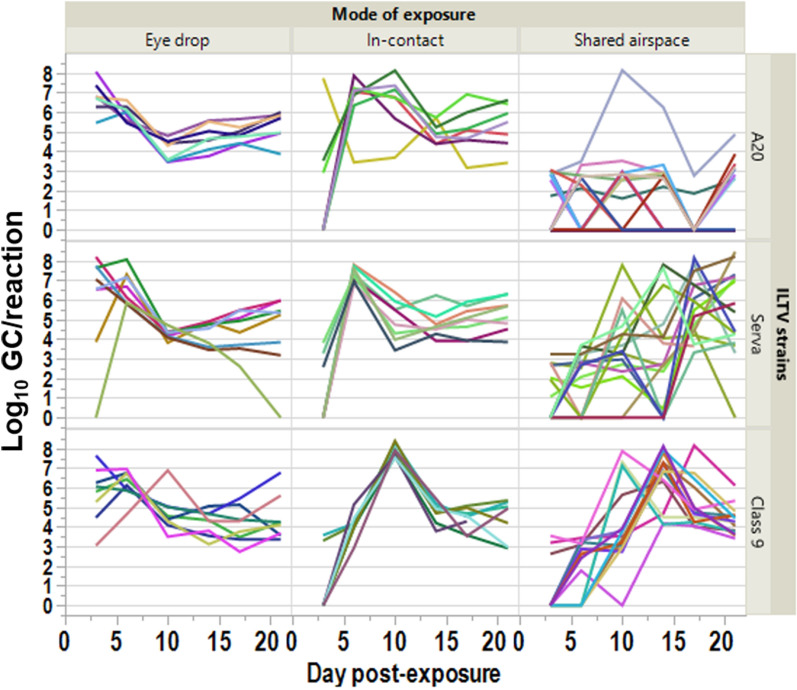
**Experiment 1 (CONTACT).** Individual bird profiles of ILTV GC detected in choanal cleft swabs, by ILTV strain, route of transmission and dpe.

Individual profiles of ILTV GC load in chickens in the different treatments are shown in Figure 2 and reveal marked differences in the profiles between modes of infection and ILTV strain. Eye drop infected birds mostly exhibited maximum load at the first sampling at 3 dpe with values then tending to decline by 1–4 logs to 10 dpe before varying around this level until the last sampling at 21 dpe. In-contact birds tended to have uniform profiles with a clear peak in GC load of similar magnitude for the 3 strains (approximately 10^8^/reaction) at 6 dpe for Serva, 6–10 dpe for A20 and 10 dpe for Class 9 indicating clear lags in timing of infection from the eye drop infected birds. Values then declined sharply by 2–4 logs before varying around this level until the last sampling at 21 dpe. Profiles were more varied for birds sharing an air space with infected birds with evidence of delayed transmission of the Class 9 and Serva strains to all birds, but only to some birds in the case of the A20 strain.

**Figure 3:**
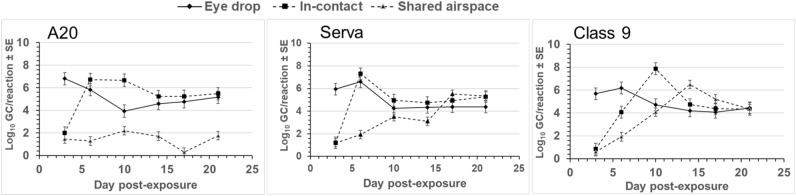
**Experiment 1 (CONTACT).** Viral load of A20, Serva and Class 9 in choanal cleft swabs (log_10_ GC/reaction, LSM ± SE) showing the interacting effects of ILTV strain, mode of exposure and dpe. Solid lines represent the eye drop inoculated birds and the dashed lines represent the in-contact and shared airspace exposed birds.

The results of formal analysis of variance of the swab qPCR data are summarised in Table 3. The proportions of birds positive for ILTV DNA in choanal cleft swabs and the ILTV GC load in swabs were significantly influenced by ILTV strain, mode of infection and dpe and in the case of clinical scores, significant interaction between these main effects (Table 3). Figure 3 illustrates the significant interaction between the effects of ILTV strain, mode of exposure and dpe on the level of ILTV GC. This clearly illustrates the lags in viral load for the in-contact and shared air space birds and the very low level of A20 vaccine virus detected in birds sharing an airspace with infected birds (Figure 3). Plasma collected from eye drop inoculated chickens was also tested by qPCR and ILTV DNA was detected from all Class 9 and Serva inoculated chickens but only a single A20 inoculated chicken.

#### Transmission of ILTV between chickens

**Figure 4:**
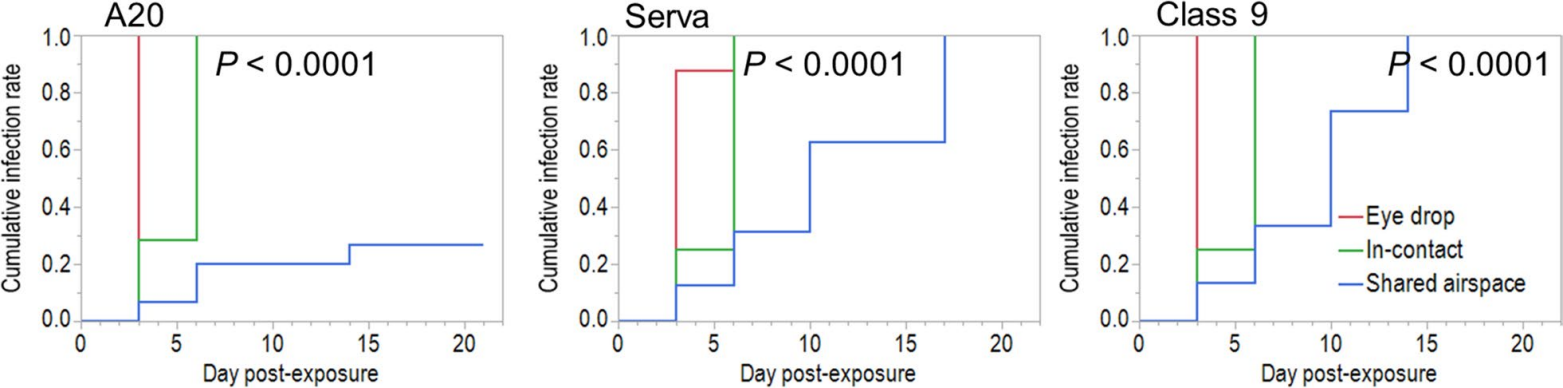
**Experiment 1 CONTACT.** Proportion of chickens becoming infected with ILTV strains, A20, Serva and Class 9 over time following different modes of exposure. *P* values are based on Kaplan–Meier survival analysis.

Based on the criteria to determine effective ILTV transmission, all birds in-contact with eye drop inoculated birds became infected with ILTV by 6 dpe regardless of ILTV strain (Table 5, Figure 4). However there was a marked difference in the transmission of the three ILTV strains to birds sharing a common airspace with infected birds (Table 5). The A20 vaccine strain transmitted poorly under these conditions to only 4/15 (27%) birds whereas the Class 9 and Serva strains transmitted to 100% of chickens by 14 and 17 dpe respectively (Figure 4).

**Table 5:** **Experiment 1 CONTACT. Proportion of birds becoming infected with ILTV following exposure to A20, Serva and Class 9 ILTV by eye drop inoculation, contact with inoculated birds or sharing an airspace with inoculated in the 21 days post exposure**

ILTV strains	Proportion of chickens infected with ILTV (%)*			
	Eye drop	In-contact	Shared airspace	Total
A20	7/7 (100)^a^	7/7 (100)^a^	4/15 (27)^bA^	18/29 (62)^A^
Serva	8/8 (100)	8/8 (100)	16/16 (100)^B^	32/32 (100)^B^
Class 9	8/8 (100)	8/8 (100)	15/15 (100)^B^	31/31 (100)^B^
Total	23/23 (100)	23/23 (100)	35/46 (76)	81/92 (88)
Factor				*P* value
ILTV strain				< 0.0001
Mode of exposure				0.0019

Proportion of birds becoming infected with ILTV following exposure to A20, Serva and Class 9 ILTV by eye drop inoculation, contact with inoculated birds or sharing an airspace with inoculated in the 21 dpe.

^*^As defined in “Materials and methods” section.

^ab^Different lowercase superscripts indicate significant differences within rows.

^AB^Uppercase superscripts indicate a significant differences within columns for each ILTV strain. (*P* < 0.05).

### Experiment 2. Dust infectivity (DUST INF)

#### Generation of dust containing Class 9 ILTV

Birds in 4 isolators were successfully eye drop infected with Class 9 virus to generate dust from infected chickens. The most common clinical sign observed in these birds was conjunctivitis. The birds were assessed for ILTV infection by qPCR of choanal cleft swab. Of 20 chickens sampled on each of days 3, 7, 10 and 14 by choanal cleft swab 11 (55%), 19 (95%), 20 (100%) and 20 (100%) were positive for ILTV infection indicating successful eye drop infection with Class 9. The highest log_10_ ILTV GC/reaction were recorded at 7 dpe (6.1 ± 0.38) and 10 dpe (3.81 ± 0.38). The ILTV GC load in dust samples collected on days 3, 7 and 14 for use in the dust transmission experiment are reported in Section “Experiment 2. Dust infectivity (DUST INF)”.

#### Transmission of ILTV by inoculation with dust or dust extracts

No clinical signs of ILTV or mortality were observed following inoculation with dust extracts or dust from infected chickens. All choanal cleft swabs at 7 (*n* = 82) and 14 (*n* = 40) dpe were negative for ILTV DNA by qPCR indicating absence of transmission of ILTV by qPCR positive dust from infected birds.

### Experiment 3. Infectivity of blood and plasma from ILTV infected chickens (BLOOD INF)

None of the birds inoculated with blood (intra-coelomic) or plasma (eye drop) collected from birds with active infection with field (Class 9) and vaccine (Serva and A20) ILTV showed clinical signs of ILT and analysis of qPCR of choanal cleft swabs at 7 and 14 dpe were negative for transmission of ILTV.

## Discussion

In the present experiment, direct contact between chickens was shown to enable rapid transmission of both virulent and vaccine strains of ILT, but sharing a common airspace without direct contact between birds greatly slowed the rate of transmission, particularly for the A20 vaccine virus. To the best of our knowledge this is the first such comparison and builds on our earlier study on airborne transmission [24]. Despite the clear evidence of airborne transmission in the present experiment, we were unable to infect chickens with fresh dust or extracts of fresh dust from ILTV infected chickens, calling into question the importance of dust particles in airborne transmission. Similarly, infectivity of fresh blood and/or plasma from ILTV infected chickens was not demonstrated, despite detection of ILTV GC in such samples.

The first proposition that field and vaccine ILTV strains will transmit much more effectively to in-contact susceptible birds than those sharing an air space without direct contact was supported by the results. For birds in-contact with those infected with the three ILTV strains 100% of infection was recorded by 6 dpe in all cases. Similarly, 100% transmission of Serva vaccine strains was reported by 8 dpe for in-contact birds housed with birds infected via drinking water and eye drop [22]. In another study, Coppo et al. [21] reported 50–100% transmission of Serva to in-contact birds between 4–8 dpe while only 25% of birds in-contact with SA2 birds inoculated via drinking water became infected by 12 dpe [21].

As predicted, prevention of direct contact with inoculated birds greatly slowed the rate of transmission of the 3 ILTV strains with Class 9 and Serva strains transmitting to 100% of chickens by 14 and 17 dpe respectively while the A20 vaccine strain transmitted poorly to only 27% by 21 dpe. This indicates that although airborne transmission slows the spread of ILTV it was still effective enough to ensure 100% infection for two of the strains. These results confirm the airborne route of transmission reported by Yegoraw et al. [24] in which air was passed from infected to susceptible birds through a 2 m hose duct. In that experiment the same 3 ILTV strains were included, with somewhat lower transmission rates recorded (Class 9, 67%, Serva and A20, 30%). These studies are the first to demonstrate airborne transmission of ILTV, although it has long been postulated. The much more rapid transmission of ILTV strains when birds are in-contact with each other, may simply reflect the shorter distance beween chickens with reduced opportunity for viral dilution and inactivation in the environment between birds shedding the virus and those inhaling it. The lower rate of airborne transmission seen when air was passed through a 2 m hose in the earlier study provides some support for this. However, it is also possible that virus is passed directly between birds by physical contact with exudates from the eye and respiratory tract. The infectivity of such materials and the persistence of the ILTV in them is well documented [48, 49] and their potential for transmission of the virus between flocks on fomites recognised.

Previous in vivo studies have shown significant variation in replication kinetics, pathogenicity, infectivity and transmissibility to in-contact birds between different ILTV strains [15, 50, 51]. This is supported by the findings of the present study which showed that the two vaccine strains transmitted and/or replicated more rapidly to in-contact birds than the virulent strain, having a peak ILTV load at 6 dpe compared to 10 days for the virulent strain. However, when direct contact was prevented and birds shared a common airspace, the highest log_10_ ILTV genome load was observed for the virulent virus (3.76 ± 0.19) followed by the vaccine viruses Serva (3.42 ± 0.21) and A20 (0.31 ± 0.24). Similarly, in the previous airborne transmission study, higher viral load was recorded in birds exposed to air from Class 9 infected birds than the vaccine strains [24]. These data are suggestive of strain differences in mode of transmission between the Australian ILTV strains with vaccine viruses transmitting very effectively to in-contact birds while virulent virus is more effective at airborne transmission. However, Groves et al. [6] reported comparatively slow spread of the Serva vaccine virus to in-contact birds in large commercial poultry houses following sub-optimal initial vaccine take indicating that effective spread between chickens should not be relied upon to compensate for sub-optimal mass vaccination methods. The difference between these findings in the field and our experimental results may be due to the vast difference in population sizes involved in the two studies and also the different route of infection used for the primary infection. In the field study mass vaccination was via the water system, while in the present study it was by eye drop, and as speculated above, eye exudates may play an important role in transmission by direct contact between chickens.

Given the demonstrated airborne transmission of ILTV [24] and implication of spread associated with spreading of spent litter or movement of vehicles carrying infected chickens [25] our second proposition was that fresh dust and dust extract from ILTV infected birds will be infective to susceptible chickens. However, this was not supported by the findings in which infection was unsuccessful with dust or dust extracts collected from infected and clinically sick birds at 3, 7 and 14 dpe and given to 120 susceptible chicks in 6 isolators. This is in agreement with previous report by Bindari et al. [36] that failed to infect cell cultures or chick embryos with qPCR positive dust extracts. These authors also found that spiking dust samples with cultured ILTV reduced the infectivity of the ILTV to chick embryos. These suggests that ILTV GC detected in dust doesn’t represent infective virus. Poultry dust is mainly comprised of aerosolised excreta [52, 53] and attempts at infecting chickens with excreta extracts from infected chickens have not been successful [24]. This may explain the lack of ILTV infectivity in dust samples containing high levels of ILTV genome.

The findings of this study did not support the third proposition that the virulent and vaccine ILTV strains will be successfully transmitted to susceptible chickens by intra-coelomic inoculation of fresh whole blood and eye drop administration of fresh plasma from infected chickens. This may be because the ILTV DNA detected in blood or plasma was not infective or that the inoculum contained insufficient infective virus. Given the comparatively large volumes of blood and plasma used in this study and the stage of infection of the birds from which these samples were obtained (6 and 10 days post-infection) these results indicate that it is very unlikely that haematophagous insects or mites, or iatrogenic transmission with vaccination needles or other husbandry equipment could transmit ILTV. However, ILTV was shown to survive inside and outside darkling beetles for several weeks after an ILT outbreak [44].

In conclusion, this study has confirmed a recent demonstration of airborne transmission of ILTV, including more efficient airborne transmission by virulent ILTV, using an alternative experimental model of a common airspace without physical contact between birds. The A20 vaccine transmitted very poorly by this route. Unlike the previous study this study included comparison with transmission between birds in physical contact with infected birds and this revealed that direct contact enabled much more efficient transmission of both virulent and vaccine strains with 100% transmission by 6 dpe. However, the vaccine strains appeared to replicate more rapidly following in-contact transmission achieving peak viral load at 6 dpe compared to 10 dpe for the virulent strain. These results are indicative of interaction between ILTV strain and mode of transmission with virulent virus more successful at airborne transmission and vaccine viruses more successful at in-contact transmission. Dust or extracts of dust from infected chickens were not infective to susceptible birds which is suggestive of a less important role of dust in the epidemiology of ILTV than previously speculated. Similarly, blood and plasma from infected birds were not infective which is supportive of earlier reports of a lack of detected viraemia in infected birds.

## Abbreviations

CEO: Chick embryo origin
DNA: Deoxyribonucleic acid
dpe: Days post-exposure
EID: Embryo infective dose
GC: Genome copies
ILT: Infectious laryngotracheitis
ILTV: Infectious laryngotracheitis virus
LSM: Least squares means
NSW: New South Wales
PCR: Polymerase chain reaction
PFU: Plaque forming unit
qPCR: Quantitative polymerase chain reaction
SE: Standard error
TCID: Tissue culture infective dose
